# Anterior Cervical Discectomy with Arthroplasty versus Arthrodesis for Single-Level Cervical Spondylosis: A Systematic Review and Meta-Analysis

**DOI:** 10.1371/journal.pone.0043407

**Published:** 2012-08-17

**Authors:** Aria Fallah, Elie A. Akl, Shanil Ebrahim, George M. Ibrahim, Alireza Mansouri, Clary J. Foote, Yuqing Zhang, Michael G. Fehlings

**Affiliations:** 1 Division of Neurosurgery, University of Toronto, Toronto, Canada; 2 Department of Clinical Epidemiology and Biostatistics, McMaster University, Hamilton, Canada; 3 Department of Medicine, State University of New York at Buffalo, Buffalo, New York, United States of America; 4 Institute of Medical Sciences, University of Toronto, Toronto, Canada; 5 Division of Orthopedic Surgery, McMaster University, Hamilton, Canada; 6 Guang'anmen Hospital, China Academy of Chinese Medical Science, Beijing, China; 7 Toronto Western Research Institute, Toronto Western Hospital, Toronto, Canada; 8 Krembil Neuroscience Center, University Health Network, Toronto, Canada; The Ohio State University Medical Center, United States of America

## Abstract

**Objective:**

To estimate the effectiveness of anterior cervical discectomy with arthroplasty (ACDA) compared to anterior cervical discectomy with fusion (ACDF) for patient-important outcomes for single-level cervical spondylosis.

**Data sources:**

Electronic databases (MEDLINE, EMBASE, Cochrane Register for Randomized Controlled Trials, BIOSIS and LILACS), archives of spine meetings and bibliographies of relevant articles.

**Study selection:**

We included RCTs of ACDF versus ACDA in adult patients with single-level cervical spondylosis reporting at least one of the following outcomes: functionality, neurological success, neck pain, arm pain, quality of life, surgery for adjacent level degeneration (ALD), reoperation and dysphonia/dysphagia. We used no language restrictions. We performed title and abstract screening and full text screening independently and in duplicate.

**Data synthesis:**

We used random-effects model to pool data using mean difference (MD) for continuous outcomes and relative risk (RR) for dichotomous outcomes. We used GRADE to evaluate the quality of evidence for each outcome.

**Results:**

Of 2804 citations, 9 articles reporting on 9 trials (1778 participants) were eligible. ACDA is associated with a clinically significant lower incidence of neurologic failure (RR  = 0.53, 95% CI  = 0.37–0.75, p = 0.0004) and improvement in the Neck pain visual analogue scale (VAS) (MD  = 6.56, 95% CI  = 3.22–9.90, p = 0.0001; Minimal clinically important difference (MCID)  = 2.5. ACDA is associated with a statistically but not clinically significant improvement in Arm pain VAS and SF-36 physical component summary. ACDA is associated with non-statistically significant higher improvement in the Neck Disability Index Score and lower incidence of ALD requiring surgery, reoperation, and dysphagia/dysphonia.

**Conclusions:**

There is no strong evidence to support the routine use of ACDA over ACDF in single-level cervical spondylosis. Current trials lack long-term data required to assess safety as well as surgery for ALD. We suggest that ACDA in patients with single level cervical spondylosis is an option although its benefits and indication over ACDF remain in question.

## Introduction

### Rationale

Cervical spondylosis is a common cause of radiculopathy and/or myelopathy resulting in significant disability [1]. In patients that do not respond adequately to conservative management, anterior cervical discectomy with fusion (ACDF) is performed to achieve neural decompression, maintain cervical lordosis and provide segmental stabilization. ACDF halts neurological deterioration and relieves radicular symptoms in patients with myelopathy and radiculopathy, respectively. However, fusion results in increased biomechanical forces at the adjacent (mobile) level and may thus accelerate symptomatic degenerative progression [2]; some of these patients may require further surgery at the adjacent level.

Anterior cervical discectomy with arthroplasty (ACDA) is an alternative surgical option that could preserve segmental mobility at the diseased level and theoretically decrease the incidence of adjacent level degeneration (ALD). The key difference in this procedure compared to an ACDF is a wider decompression (i.e. generous bilateral foraminotomies) including resection of the uncovertebral joints bilaterally. Further, patients are commonly prescribed non-steroidal anti-inflammatory medication to prevent heterotopic ossification in addition to postoperative pain control. Heterotopic ossification is most commonly described as a complication of large joint arthroplasty and is the main cause of the prosthesis to lose function [3]. Its prevalence in cervical arthroplasty is 58.2% (95% CI  = 29.7–86.8%) 12 months after surgery [3]. In addition, ACDA is a technically more difficult operation to perform compared to an ACDF.

**Figure 1:**
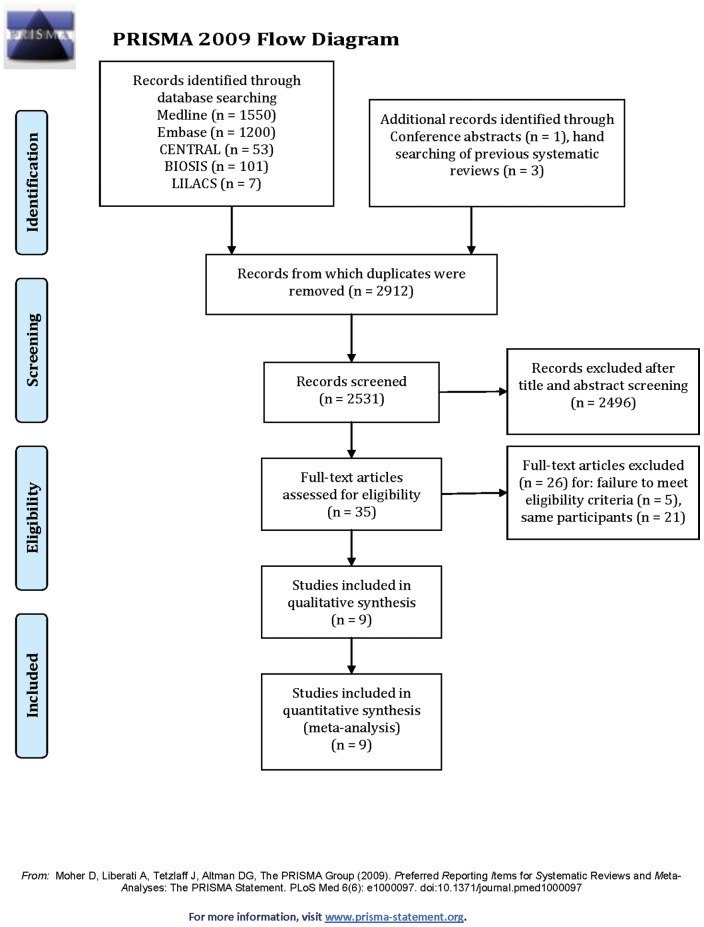
PRISMA 2009 Flow Diagram.

**Table 1:** Patient and study characteristics of the 9 included studies.

Source	Study location	Age range (years)	Main inclusion criteria	No. of patients (ACDA group)	No. of patients (ACDF group)	Prosthetic device	Outcomes reported	Minimal follow-up duration (months)	No. of centers	Duration of study	Type of RCT/Expertise based design?	Superiority, non-inferiority or equivalence trial	Protocol registered?	Surgical procedure described?
Burkus et al. 2010[23]	USA	>18	Single level symptomatic DDD, C3-7, NDI scores ≥30	144	127	Prestige	a; b; c; d; e; f; g; h	60	31	2002–2004	Parallel/Yes	Not mentioned	No	Yes
Coric et al. 2011[6]	USA	18–60	Single level symptomatic DDD, C3-7, NDI scores ≥40	136	133	Kineflex-C	a; c; d**; e**; f; g; h; i	24	21	N/A	Parallel/No	Non-inferiority	Yes	Yes
Delamarter et al. 2010[25]	USA	18–60	Single level symptomatic DDD, C3-7, NDI scores ≥15	103	106	ProDisc-C	a; b; c; d; e; g; h	48	13	2003–2004	Parallel/No	Non-inferiority	No	Yes
McAfee et al. 2011* [21]	USA	18–65	Single level symptomatic DDD, C3-T1, NDI ≥30	188	151	PCM, Bryan, Prestige and ProDisc-C*	a; c	24	N/A	N/A	Parallel/N/A	Not mentioned	No	No
McAfee et al. 2010[20]	USA	18–65	Single level symptomatic DDD, C3-T1, NDI ≥30	151	100	PCM	f	24	5	N/A	Parallel/N/A	Not mentioned	No	No
Nabhan et al. 2007[24]	Germany	20–60	Single level symptomatic (Radiculopathy only) DDD, C3-7	20	21	ProDisc-C	d; e	36	1	2004–2005	Parallel/No	Not mentioned	No	Yes
Nabhan et. al 2011[26]	Germany	N/A	Symptomatic cervical (radiculopathy only) DDD	10	10	ProDisc-C	a; d; e	12	1	2006–2007	Parallel/No	Not mentioned	No	Yes
Sasso et. al 2011[5]	USA	≥21	Single level cervical symptomatic DDD	181	138	Bryan	a; b; c; d; e; g; h;	48	30	2002–2004	Parallel/No	Superiority	Yes	No
Wang et. al 2008[22], [25]	China	30–50	Single level symptomatic DDD, C3-7	28	31	Bryan	a; d; e	24	1	2003–2005	Parallel/No	N/A	No	Yes

* This article is a meta-analysis and the only study that reports the results of the PCM disc trial. Only data regarding the PCM disc trial was extracted.

** Neck and Arm VAS pain score is combined.

Outcomes reported: a  =  NDI; b  =  SF-36 PCS; c  =  Neurological Success; d  =  Neck VAS pain score; e  =  Arm VAS pain score; f  =  dysphagia/dysphonia; g  =  Operations for ALD; h  = Repeat operations; i  =  length of hospital stay.

LOCF – Last outcome carried forward.

If ALD is truly decreased, this procedure may result in decreased disability, decreased incidence of reoperation and increased quality of life while achieving similar rates of neurological success. If not, the use of ACDA increases health care costs without any additional neurological benefit [4] and a potential of greater harm if performed by a non-expert surgeon. Further, the long-term risks associated with ACDA may not be as well delineated compared to the more commonly performed ACDF.

**Figure 2:**
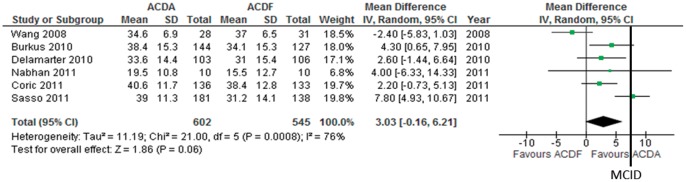
Neck disabiltiy index improvement in participants undergoing ACDA vs. ACDF for single level cervical spondylosis. CI indicates confidence interval.

**Figure 3:**
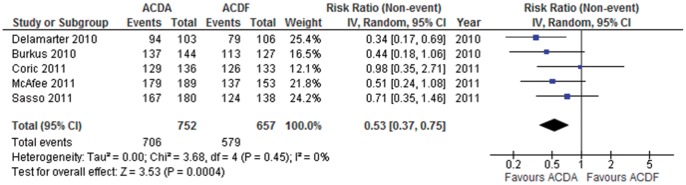
Neurological success in participants undergoing ACDA vs. ACDF for single level cervical spondylosis. CI indicates confidence interval.

Although several randomized clinical trials (RCTs) have compared ACDF to A [5]–[9], it remains unclear whether ACDA results in improved patient-important outco [4], [10] and whether or not its widespread use should be advocated. A systematic review found that ACDA results in modest clinical benefits with respect to neck pain, arm pain and quality of life compared to ACDF at 12 month follow-up, most of which were not sustained at 2 year follow-up [4]. A recent review of 3 United States Food and Drug Administration cervical arthroplasty trials concluded that ACDA may be associated with a higher rate of neurological success and lower prevalence of ALD 2 years following surgery [10].

**Figure 4:**
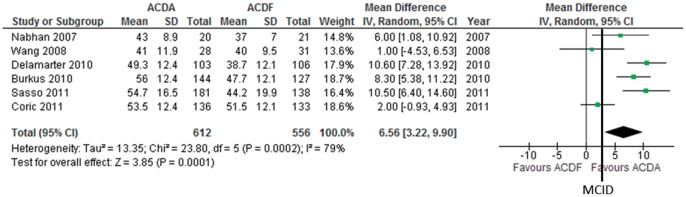
Neck visual analogue scale pain score improvement in participants undergoing ACDA vs. ACDF for single level cervical spondylosis. CI indicates confidence interval.

**Figure 5:**
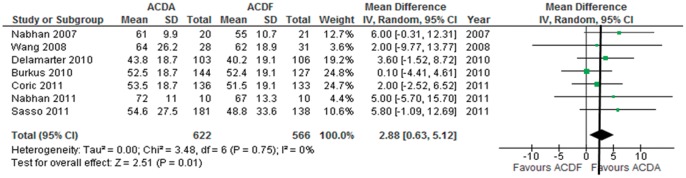
Arm visual analogue pain score improvement in participants undergoing ACDA vs. ACDF for single level cervical spondylosis. CI indicates confidence interval.

There are no systematic reviews that have assessed publication bias, evaluated the risk of bias of included trials, interpreted the results with respect to clinical significance, evaluated the quality of the evidence using the GRADE approach [11] (this is a systematic and explicit method to evaluate the quality of the evidence), and reported review findings in concordance with PRISMA guidelines [12]. This review will improve upon the methodological shortcomings of the previous studies as well as include recently published trials.

**Figure 6:**
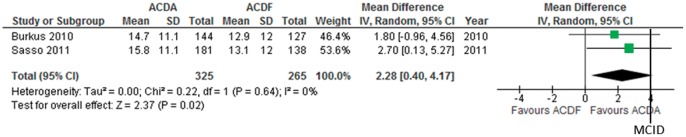
SF-36 physical component summary score improvement in participants undergoing ACDA vs. ACDF for single level cervical spondylosis. CI indicates confidence interval.

**Figure 7:**
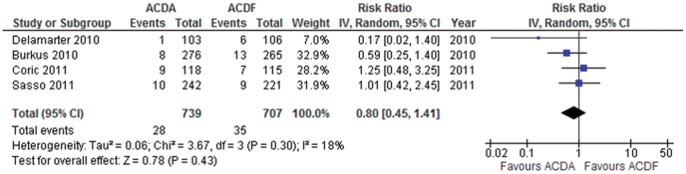
Surgery for ALD in participants undergoing ACDA vs. ACDF for single level cervical spondylosis. CI indicates confidence interval.

### Objective

We systematically reviewed all randomized clinical trials comparing the relative effects of ACDF to ACDA for single-level cervical spondylosis on patient-important outcomes.

## Methods

### Protocol and registration

We developed a protocol prior to conduct of the review but did not register it.

### Eligibility criteria

Eligible studies had to include adult patients (greater than 50% over 19 years of age at the time of inclusion), with single-level cervical spondylosis (i.e. C3-T1) causing radiculopathy and/or myelopathy, who have undergone single-level ACDF or ACDA. Our outcomes of interest were the following: functionality, pain, quality of life, surgery for ALD, reoperations, and dysphonia/dysphagia. We only included RCTs. We excluded articles that were duplicate reports of an earlier trial, post-hoc analysis of RCT data or those in which we were unable to obtain the full-text article.

**Figure 8:**
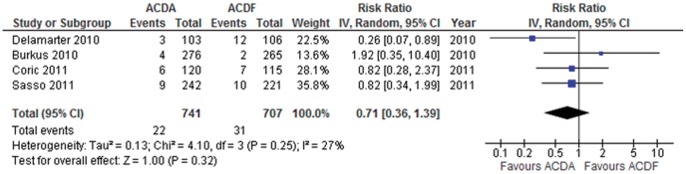
Reoperation in participants undergoing ACDA vs. ACDF for single level cervical spondylosis. CI indicates confidence interval.

**Figure 9:**
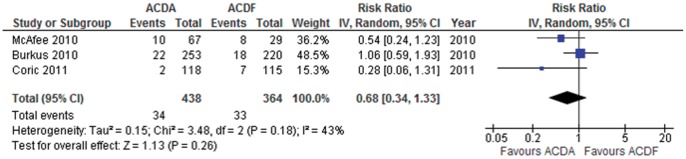
Dysphonia/dysphagia in participants undergoing ACDA vs. ACDF for single level cervical spondylosis. CI indicates confidence interval.

### Information sources

We searched MEDLINE (2002-January 2012), Embase (2002-January 2012), Cochrane Central Register of Controlled Trials (Issue 1 of 12, Jan 2012), BIOSIS (2002-January 2012) and LILACS (2002-January 2012). We restricted the search to humans and adults (19 years of age and older) but not to any specific language(s). We limited the search to 2002 onwards because the earliest trial included in a previously published systematic review was published in 2004 [13]. We imported all search results into Endnote X5 for removal of duplicates, and title and abstract screening.

**Figure 10:**
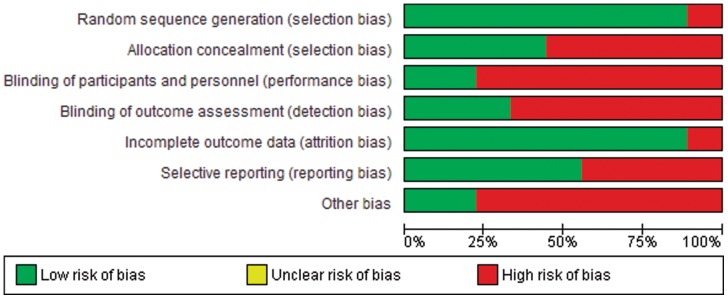
Cochrane risk of bias across studies.

**Figure 11:**
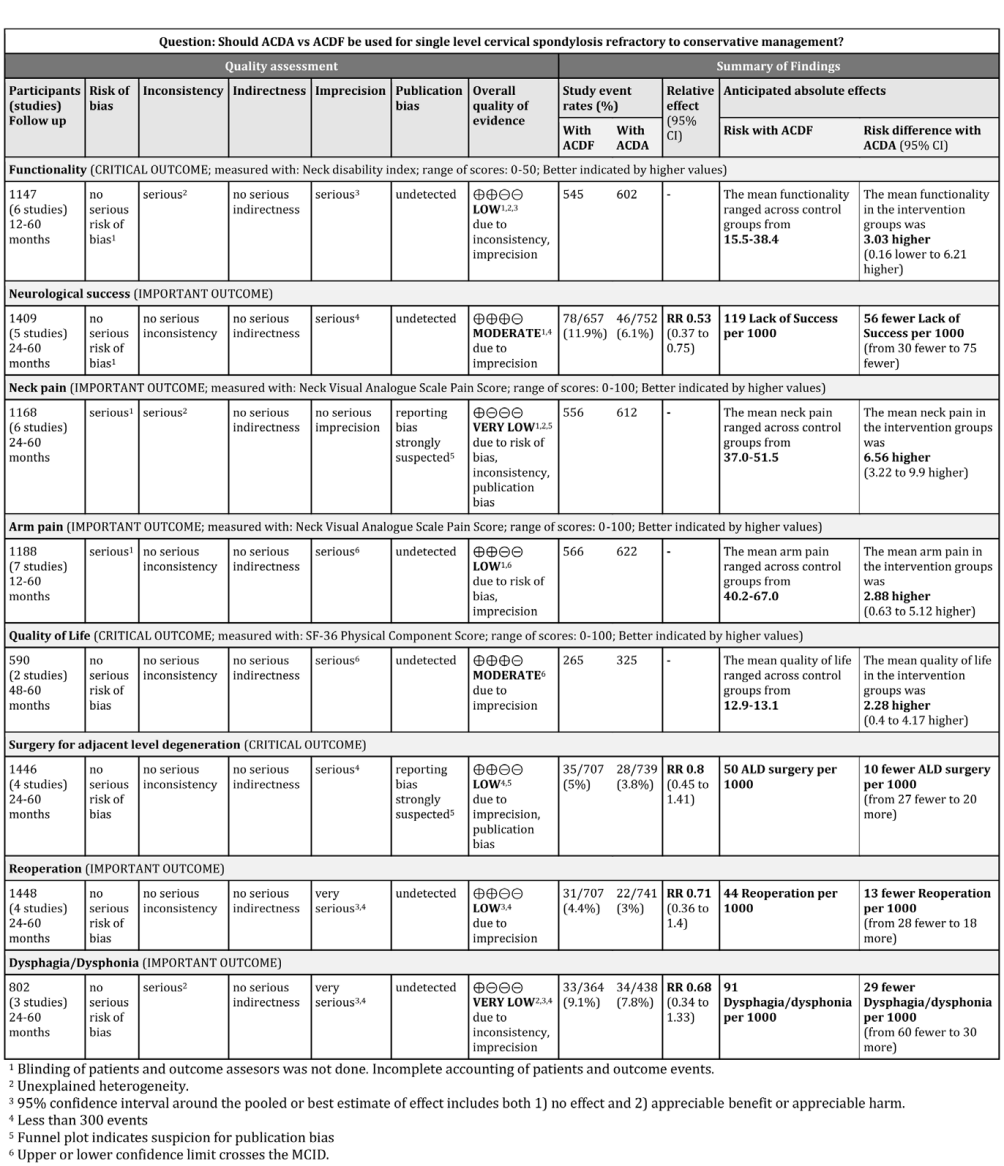
Grade profile for ACDA vs. ACDF for single level cervical spondylosis.

We hand searched reference lists of included articles and conference abstracts for the 2011 AANS/CNS Section on Disorders of the Spine and Peripheral Nerves and Eurospine 2011 meeting. We translated non-English papers. The first author (A.F.) designed and conducted the search strategy with reference to a systematic review on this topic [4] and reference to a highly sensitive search strategy for identifying randomized trials [14].

### Search

We used the following search terms in Ovid MEDLINE(R) and Embase: ‘cervical spondylosis’, ‘cervical vertebrae’, ‘prosthesis’, ‘discectomy’, ‘arthroplasty’, ‘spinal fusion’, ‘randomized-controlled trial’, ‘random allocation’ and ‘clinical trials’ (Appendix S1). We used the term “cervical arthroplasty” to search the Cochrane Central trials registry, BIOSIS and LILACS.

### Study selection

#### Title and abstract screening

Three reviewers (A.F., S.E. and G.M.I.) screened independently and in duplicate, the title and abstracts of identified citations for potential eligibility; we obtained the full text of these citations.

#### Full text review

Using a standardized form, the same reviewers independently and in duplicate applied the eligibility criteria to full text articles. We checked agreement and resolved disagreements through discussion. We calculated kappa scores to measure the degree of agreement. If articles reported on the same trial, we included the article with the most recent results or the greater number of patients. We performed calibration exercises and maintained a full list of excluded articles with reasons for exclusion.

We performed title and abstract screening, full text review and data abstraction in ‘RefWorks’ and ‘Endnote X5’ softwares.

#### Grading the quality of evidence

Two reviewers (A.F. and G.M.I.) independently and in duplicate, applied GRADE to eligible trials. The instructional manual in the ‘GRADEprofiler’ software version 3.6 was utilized to guide ratings. We downgraded the quality of evidence only by 1 for each component. Risk of bias was assessed as serious for subjective outcomes (i.e. neck pain and arm pain) when blinding was not performed. Inconsistency was determined by an I^2^ value of greater than 40% which could not be explained by our predefined subgroup analysis. We marked down for imprecision if the estimate crossed the nil effect point, unless the 95% CI did not cross the MCID (for continuous outcomes). We marked down for publication bias if this was suspected by visual inspection of the funnel plot.

### Data collection process

We developed and pilot-tested a data extraction form on an electronic spreadsheet. One reviewer (A.F.) extracted data from the included trials while a second reviewer (A.M.) checked the extracted data for accuracy. We resolved disagreements through discussion.

### Data items

Reviewers extracted the following information from each included trial: (1) characteristics of the trial (including number of trial centers, year of trial, type of RCT, trial location, length of follow-up and eligibility criteria); (2) characteristics of trial participants (number and mean age of participants in each trial arm); (3) name of prosthetic device utilized and whether the surgical procedure was described; (4) Outcomes of interest; (5) Cochrane risk of bias characteristics as well as other characteristics that may lead to bias (A priori registration of protocol, expertise based trial design, funding sources, method of statistical analysis (i.e. intention to treat or per protocol analysis) and affiliation of the authors with the medical device company).

### Risk of bias in individual studies

To ascertain the validity of the included randomized trials, reviewers independently determined the adequacy of randomization; concealment of allocation; blinding of participants, providers, outcome assessors, data collectors and data analysts; the extent of loss to follow-up; freedom from selective outcome reporting; and freedom from other bias [15] (this was used to assess whether the trial authors were affiliated or the trial was funded with the prosthesis company) [16]. We used a ‘Modification of Cochrane Tool to assess risk of bias in randomized trials’ where a forced decision regarding bias must be made into ‘probably no’ or ‘probably yes’ for items that are thought to be of unclear risk [17], [18] (Appendix S1). We judged trials with more than 2 and more than 4 high risk components as moderate risk and high risk, respectively.

### Summary measures

For continuous data, we calculated the pooled mean difference (MD) and its 95% CI using the change from baseline scores, standard deviation (SD) and total number of participants in each treatment arm. If the SD was not reported, we imputed this from a reported p value, confidence interval (CI) or standard error. In cases where none of these were available, we used the mean SD from other trials. For binary outcomes, we calculated the relative risk (RR) and its 95% CI using the number of events and total participants in each treatment arm. To facilitate interpretability, we converted RR ratios to absolute risk reductions (ARR) and number needed to treat (NNT). We selected values for minimal clinically important differences (MCID) through a literature review. MCID values were selected based on the methodological rigour of the study and the similarity of the patients to this review.

### Planned method of analysis

Any kind of variability across trials in a systematic review is termed heterogeneity. Variability may result from clinical diversity (i.e. the participants or interventions differ across trials) or methodological diversity (i.e. methodological design and risk of biases differ across trials). When the statistical tests for heterogeneity (variability in the treatment effects between trials) is significant, this is unlikely to be attributed to chance alone [19]. We explored heterogeneity using the I^2^ statistic. This statistic aims to assess the impact of the heterogeneity on the meta- analysis [19]. We considered an I^2^ score of 0% to 40% as “heterogeneity might not be important”; 30% to 60% as “may represent moderate heterogeneity*; 50% to 90% as “may represent substantial heterogeneity” and; 75% to 100% as “considerable heterogeneity” [19]. We explored heterogeneity greater than 30% by performing a priori specified subgroup analyses: type of prosthesis used and the length of follow-up (24 months versus less than 24 months). We utilized a random effects (inverse variance) model to account for heterogeneity amongst trials.

### Risk of bias across studies

We assessed the possibility of publication bias by evaluating funnel plots. A funnel plot is a scatter plot of the intervention effect estimates from individual trials against a measure of its precision [20]. In the absence of publication bias, the plot will resemble an inverted funnel. Although there are several reasons for asymmetric funnel plots, its presence generally indicates publication bias or is due to exaggeration of treatment effects in small, low quality trials [20].

### Additional analyses

We performed sensitivity analyses for any continuous outcome with a statistically significant result (and greater than the MCID where applicable) for which SD was estimated; in these cases, we assumed the highest and lowest SD from other trials to determine the robustness of our conclusions.

## Results

### Study selection

Figure 1 illustrates the study flow. We identified a total of 9 articles reporting on 9 trials (1778 participants) for inclusion (Table 1). Appendix S1 presents the list of excluded articles with reasons for exclusion. We achieved excellent agreement for screening of full text articles (Kappa  = 0.92, SE 0.08; 95% CI  = 0.78–1.07).

### Study characteristics

Tables 1 and Figure 11 present the study and participant characteristics of the included trials.

### Risk of bias within studies

Appendix S1 presents the Cochrane risk of bias assessment of the included articles. There were 3 trials with high risks of bias [20]–[22], 3 trials with moderate risk of bias [5], [23], [24], and 3 trials with low risk of bias [6], [25], [26] (Appendix S1).

### Results of individual studies and synthesis of results

#### Functionality

Six trials consisting of 1147 pariticpants report on NDI change (continuous outcome) following surgery to measure functionality. We imputed the SD using the p value in 1 trial [23], the 95% CI in 1 trial [5], the SD of baseline and end scores in 3 trials [22], [25], [26] and the mean SD in 1 trial [6]. Pooled analysis shows that ACDA is associated with a greater improvement in NDI compared to ACDF: MD (95% CI)  = 3.03 (−0.16 to 6.21), p = 0.06 (Figure 2). The upper and lower limits of the CI are smaller than the minimally clinically important difference (MCID) of 7.5 [27] and 8.5 [28] identified in the literature. There is considerable heterogeneity (I^2^ = 76%) which can not be explained using our predefined subgroup analysis. The quality of evidence for this outcome is low.

#### Neurological Success

Five trials consisting of 1409 participants report on neurological success (dichotomous outcome). Pooled analysis shows that ACDA is associated with a higher incidence of neurological success compared to ACDF: RR (95% CI)  = 0.53 (0.37 to 0.75), p = 0.0004 (Figure 3). Heterogeneity amongst the trials might not be important (I^2^ =  0%). This translates into an ARR of 5.8% (NNT  = 17). The quality of evidence for this outcome is moderate.

#### Neck pain

Six trials consisting of 1168 participants report on neck pain using the Visual Analogue Scale (VAS) (continuous outcome). We imputed unreported SD using the 95% CI in 1 trial [5], the SD of baseline and end scores in 2 trials [22], [24] and the mean SD in 3 trials [6], [23], [25]. One trial reports neck and arm pain together [25]. Pooled analysis shows that ACDA is associated with a greater improvement in neck pain compared to ACDF: MD (95% CI)  = 6.56, (3.22 to 9.90), p = 0.0001 (Figure 4). This effect is greater than the MCID of 2.5 [27]. There is moderate heterogeneity (I^2^ = 79%) which can not be explained using our predefined subgroup analysis. The quality of evidence for this outcome is very low.

#### Arm pain

Seven trials consisting of 1188 paritcipants report on arm pain using the VAS (continuous outcome). The SD was imputed using the 95% CI in 1 trals [5], the SD of baseline and end scores in 2 trials [24], [26] and the mean SD in 3 trials [6], [23], [25]. One trial reports neck and arm pain together [25]. Pooled analysis shows that ACDA is associated with a greater improvement in arm pain on the VAS compared to ACDF: MD (95% CI)  = 2.88 (0.63 to 5.12), p = 0.01 (Figure 5). However, the 95% CI of the summary effect crosses the MCID threshold of 2.5 [27]. Heterogeneity amongst the trials might not be important (I^2^ =  0%). The quality of evidence for this outcome is low.

#### Quality of life

Two trials consisting of 590 participants report on the SF-36 physical component score (PCS) change (continuous outcome) following surgery to measure quality of life. The SD is not available in one trial but was estimated using the SD in the other trial [23]. Pooled analysis shows that ACDA is associated with a greater improvement in SF-36 PCS compared to ACDF: MD (95% CI)  = 2.28 (0.40 to 4.17), p = 0.02 (Figure 6). However, the 95% CI of the summary effect spans the MCID threshold of 4.1 [27]. Heterogeneity amongst the trials might not be important (I^2^ = 0%). The quality of evidence for this outcomes is moderate.

#### Surgery for adjacent level disease

Four trials consisting of 1446 participants report data on surgery for ALD (dichotomous outcome). Pooled analysis shows that participants that undergo an ACDA are at a non-statistically significant lower risk to undergo surgery for ALD in comparison to those that undergo ACDF: RR (95% CI)  = 0.80 (0.45 to 1.41) p = 0.43 (Figure 7). This translates to an ARR of 1.2% (NNT  = 83). Heterogeneity amongst the trials might not be important (I^2^ = 18%). The quality of evidence for this outcome is low.

#### Reoperation

Four trials consisting of 1448 participants reported on reoperation (dichotomous outcome). Pooled analysis shows that participants that undergo an ACDA are at a non-statistically significant lower risk to undergo a reoperation in comparison to those that undergo ACDF: RR (95% CI)  = 0.71 (0.36 to 1.39) p = 0.32 (Figure 8). This translates to an ARR of 1.4% (NNT  = 71). Heterogeneity amongst the trials might not be important (I^2^ = 27%). The quality of evidence for this outcome is low.

#### Dysphonia/Dysphagia

Three trials consisting of 802 participants report on surgery for dysphonia/dysphagia. The trial by McAfee et al. only includes participants with dysphagia [20]. Pooled analysis shows that participants that undergo an ACDA are at a non-statistically significant lower risk to develop dyphagia/dyphonia in comparison to those that undergo ACDF: RR (95% CI)  = 0.68 (0.34 to 1.33), p = 0.26 (Figure 9). This translates to an ARR of 1.3% (NNT  = 77). Heterogeneity amongst the trials may be moderate (I^2^ = 43%) and is not explained using our predefined subgroup analysis. The quality of evidence for this outcome is very low.

### Risk of bias across studies

The trials generally had a high risk of bias for lack of blinding and affiliation with the sponsoring implant manufacturing company, moderate risk of bias for poor allocation concealment, lack of blinding of outcome assessors and selective reporting bias, and low risk of bias for randomization sequence generation and incomplete outcome data (Figure 10). Due to the small number of trials for each outcome, we could not reliably detect publication bias. However, funnel plots were assymmetric, raising suspicion for publication bias, for ALD requiring surgery and reoperation.

### Additional analysis

#### Sensitivity analysis

Statistical heterogeneity for functionality is eliminated if the trial by Wang et al. is removed. This is the only Chinese trial and only trial that favours ACDF for functionality. Removal of this trial still results in no clinically significant improvement in functionality in comparison to ACDF.

Sensitivity analysis assuming the highest and lowest SD for estimated SD data for Neck pain VAS score improvement results in a MD (95% CI)  = 6.52, (3.24 to 9.80), p<0.0001 and MD (95% CI)  = 6.58, (3.08 to 10.07), p = 0.0002, respectively. The upper and lower threshold of the 95% CI under both assumptions is still greater than the MCID threshold of 2.5 [27].

## Discussion

### Summary and quality of evidence using GRADE

ACDA is associated with a clinically significant greater improvement in neck pain and higher incidence of neurologic success. ACDA is associated with a statistically significant but not clinically significant greater improvement in arm pain and quality of life. ACDA is associated with a non-statistically significant greater improvement in functionality, and lower incidence of ALD requiring surgery, reoperation and dysphagia/dysphonia. The quality of evidence was assessed using GRADE (Figure 11).

#### Neck pain and neurological success

It is unclear why ACDA results in a greater improvement in neck pain and neurological success as these are thought to be related to adequate neural decompression and stabilization, respectively. Perhaps, a wider lateral decompression that is required for ACDA results in a greater amount of neural decompression; this is irrespective of the spacer device used. The greater neurological success and decreased neck pain associated with ACDA should be cautiously interpreted given that the patients and outcome assessors were generally not blinded [4].

#### Functionality and surgery for adjacent level degeneration

The two most critical outcomes when considering the use of ACDA over ACDF is functionality and surgery for ALD. We obtained adequate power to conclude that there is no clinically important benefit of ACDA over ACDF. We were underpowered to determine whether ACDA results in a lower incidence of surgery for ALD. The point estimate equates to a NNT of 83. The NNT for the lower boundary of the CI equates to 37 corresponding to the most benefit one would expect to obtain. However, there are two important caveats: 1) ALD is a time dependent complication requiring studies of longer follow-up duration to determine any benefit for ACDA. Interestingly, the trial by Coric et al [6] that most strongly favoured ACDF is also the one with the shortest followup duration (i.e. 24 months) (Figure 7). A hypothesis for future studies is that any benefits of ACDA over ACDF in preventing ALD may be better appreciated in the long-term; and 2) None of the trials defined the criteria for surgical intervention for ALD a priori. Given the paucity of long-term data, there is no evidence that ACDA results in increased complications compared to ACDF. Therefore, ACDA remains an option for symptomatic single-level spondylosis.

### Comparison to other systematic reviews/meta-analysis

There are several systematic reviews and meta-analyses on this topic, some suggesting a clinical benefit of ACDA over ACDF [21], [29]–[32] while others suggested no clinical benefit of ACDA over ACDF [4], [33]. However, these systematic reviews suffer from a number of methodological limitations. Only two reviews searched for non-English articles [4], [13] and only one translated non-English articles [13]. There is evidence that trials with positive results are more likely to be published in English language journals [34]. This is particularly relevant as the only trial that found greater NDI improvement in the ACDF arm was the Chinese trial by Wang et al [22].

Cepoui-Martin et al. only qualitatively described the data [33] due to methodological flaws and poor reporting of the original trials. Although this, more conservative, approach may be appropriate, we obtained enough information and imputed data when it was unavailable to perform a meta-analysis. We tested our assumptions using sensitivity analysis. The authors of this review conclude moderate to strong evidence supporting the efficacy of ACDA but are not transparent on how GRADE was applied to formulate these recommendations. This is important as the application of GRADE has a subjective component. We graded the quality of evidence as ‘very low’ to ‘moderate’ and were transparent about our decision-making (Figure 11).

Yu et al. pooled trial results using fixed effects and concluded that neurological success, repeat operation and neck pain favour ACDA [32]. Random effects were only utilized when I^2^ was greater than 50%. Since we are pooling results from several prosthetic devices which are thought to work through different mechanisms, it is much more likely that the underlying effect is not fixed and therefore a more conservative, random effects model should be utilized.

Two reviews quantitatively pooled trials to enhance power for obtaining a statistically significant result without conducting systematic searches [21], [31]. Authors from one of these reviews had a priori knowledge that all selected trials favoured arthroplasty [21]. This approach is extremely susceptible to providing misleading results. In this review, NDI success is defined as a greater than 15 point increase; no reference is provided for a scientific basis of choosing this threshold [21].

We agree with Botelho et al. that there is a paucity of data regarding the incidence of ALD with ACDA [13]. Most trials report ALD requiring surgery which may be prone to bias if the criteria for ALD surgery are not defined a priori. The systematic review by Jiang et al. conclude that there is a lower rate of ALD surgery with ACDA [30]. However, they have included a non-randomized study [35] in their analysis, whose removal would lead to a borderline significant result.

### Strengths

There are several strengths to this meta-analysis including a rigorous search strategy, no language limitations, article screening and methodological assessments performed in duplicate, abstracted data verified by a second reviewer and utilization of the GRADE approach to summarize findings and judge the quality of evidence. In addition, this is the first systematic review on this topic to incorporate MCID in interpreting findings. This approach focuses on clinically important differences as opposed to statistically important differences.

### Limitations

There are several limitations to this meta-analysis: 1) There is a variable length of follow-up across trials. This is particularly important for evaluating surgery for ALD as this outcome is time-dependent; 2) There may be a prosthetic device that is superior to others with respect to functionality and decreased incidence of ALD. Combining data across trials may fail to identify this; 3) Reporting quality was generally poor across trials; therefore, it is unclear if a lack of difference between the 2 interventions is due to poor methodological quality of the trial or a true lack of difference in effect; and 4) We were unable to obtain data for several relevant abstracts that we identified despite contacting authors.

### Ongoing studies

We identified several ongoing trials pertaining to this topic: DISCOVER™ (NCT00432159, NCT00735176), GRANVIA®-C (NCT01518582), Mobi-C (NCT00554528) and The NeoDisc™ (NCT00478088). The eventual addition of these trials to the current body of evidence is likely to improve our confidence in the conclusions and ability to make informed treatment recommendations.

### Conclusions

#### Implications for practice

We suggest that ADCA in adult patients with single level cervical spondylosis is an option. The indication and benefits over ACDF remain in question. In addition, long-term safety data of this device is not available. We invite clinicians to consider patient's values and preferences in selecting the surgical treatment.

#### Implications for research

The quality of current evidence varied from ‘very low’ to ‘moderate’ (Figure 11). Future trials should register their protocols, not be funded or clearly be independent from influence by implant manufacturing companies [15], be adequately powered to assess all patient important outcomes, utilize an expertise-based design, safeguard against biases, centrally adjudicate indications for surgery for ALD and reoperation, fully account for all trial participants and report long-term follow-up utilizing the CONSORT guidelines.

## Supporting Information

Appendix S1 **Medline and Embase search strategy.**
(DOCX)
