# Supplementary material for: Anterior Cervical Discectomy with Arthroplasty versus Arthrodesis for Single-Level Cervical Spondylosis: A Systematic Review and Meta-Analysis
Source: PLoS One. 2012 Aug 17;7(8):e43407. doi: 10.1371/journal.pone.0043407 (PMC3422251; doi:10.1371/journal.pone.0043407)
Supplement: Appendix S1 — Medline and Embase search strategy. (DOCX) [file pone.0043407.s001.docx]

Appendix S1. Medline and Embase search strategy

OVID (Medline and Embase) Search Strategy:

--------------------------------------------------------------------------------

1 exp cervical spondylosis/ (6111)

2 cervical spondylo*.mp. (5423)

3 1 or 2 (9293)

4 exp prosthesis/ (356795)

5 exp prostheses/ (335353)

6 prosthes*.mp. (367005)

7 exp discectomy/ (7944)

8 exp diskectomy/ (7944)

9 discect*.mp. (6570)

10 4 or 5 or 6 or 7 or 8 or 9 (554211)

11 randomized-controlled trial.mp. [mp=ti, ab, sh, hw, tn, ot, dm, mf, dv, kw, nm,

ps, rs, ui] (637999)

12 randomized-clinical-trial.mp. [mp=ti, ab, sh, hw, tn, ot, dm, mf, dv, kw, nm, ps,

rs, ui] (23243)

13 randomized-controlled-trials.mp. [mp=ti, ab, sh, hw, tn, ot, dm, mf, dv, kw, nm,

ps, rs, ui] (108857)

14 random-allocation.mp. [mp=ti, ab, sh, hw, tn, ot, dm, mf, dv, kw, nm, ps, rs, ui]

(74695)

15 double-blind-method.mp. [mp=ti, ab, sh, hw, tn, ot, dm, mf, dv, kw, nm, ps, rs,

ui] (112764)

16 single-blind-method.mp. [mp=ti, ab, sh, hw, tn, ot, dm, mf, dv, kw, nm, ps, rs, ui]

(15667)

17 clinical-trial.mp. [mp=ti, ab, sh, hw, tn, ot, dm, mf, dv, kw, nm, ps, rs, ui]

(1416395)

18 exp clinical-trials/ (23705)

19 random*.mp. (1512470)

20 11 or 12 or 13 or 14 or 15 or 16 or 17 or 18 or 19 (2345876)

21 3 and 10 and 20 (183)

22 limit 21 to ("young adult (19 to 24 years)" or "adult (19 to 44 years)" or "young

adult and adult (19-24 and 19-44)" or "middle age (45 to 64 years)" or "middle aged

(45 plus years)" or "all aged (65 and over)" or "aged (80 and over)") [Limit not valid in

Embase; records were retained] (156)

23 exp arthroplasty/ (90645)

24 exp cervical vertebrae/ (50445)

25 exp intervertebral disc/ (17007)

26 exp spinal fusion/ (29588)

27 3 or 24 (56593)

28 10 or 23 or 24 or 25 or 26 (666077)

29 comparative study.mp. [mp=ti, ab, sh, hw, tn, ot, dm, mf, dv, kw, nm, ps, rs, an,

ui] (2222051)

30 multicenter study.mp. [mp=ti, ab, sh, hw, tn, ot, dm, mf, dv, kw, nm, ps, rs, an,

ui] (234731)

31 20 or 29 or 30 (4325990)

32 27 and 28 and 31 (5672)

33 limit 32 to "all adult (19 plus years)" [Limit not valid in Embase; records were

retained] (4645)

34 limit 33 to humans (4379)

35 limit 34 to yr="2002 -Current" (2748)

Appendix S2. Tool to assess risk of bias in Randomized Controlled Trials

**Tool to Assess Risk of Bias in Randomized Controlled Trials**

1. **Was the allocation sequence adequately generated?***

Definitely yes Probably yes Probably no Definitely no

(low risk of bias) (high risk of bias)

Examples of low risk of bias: Referring to a random number table; Using a computer random number generator; Coin tossing; Shuffling cards or envelopes; Throwing dice; Drawing of lots; Minimization with or without a random element.

Examples of high risk of bias: Sequence generated by odd or even date of birth; Sequence generated by some rule based on date (or day) of admission; Sequence generated by some rule based on hospital or clinic record number; Allocation by judgement of the clinician; Allocation by preference of the participant; Allocation based on the results of a laboratory test or a series of tests; Allocation by availability of the intervention.

* Option to omit this item

1. **Was allocation adequately concealed?**

Definitely yes Probably yes Probably no Definitely no

(low risk of bias) (high risk of bias)

Examples of low risk of bias allocation concealment techniques: Central allocation (including telephone, web-based, and pharmacy-controlled, randomization);

Examples of possible low risk of bias: Sequentially numbered drug containers of identical appearance; Sequentially numbered, opaque, sealed envelopes.

Examples of high risk of bias allocation generation techniques: Using an open random allocation schedule (e.g. a list of random numbers); Assignment envelopes were used without appropriate safeguards (e.g. if envelopes were unsealed or non-opaque or not sequentially numbered); Alternation or rotation; Date of birth; Case record number; Any other explicitly unconcealed procedure.

1. **Blinding: Was knowledge of the allocated interventions adequately prevented?***

Definitely yes Probably yes Probably no Definitely no

(low risk of bias) (high risk of bias)

Examples of low risk of bias: No blinding, but the review authors judge that the outcome and the outcome measurement are not likely to be influenced by

lack of blinding; Blinding of participants and key study personnel ensured, and unlikely that the blinding could have been broken; Either participants or some key study personnel were not blinded, but outcome assessment was blinded and the nonblinding of others unlikely to introduce bias.

Examples of high risk of bias: No blinding or incomplete blinding, and the outcome or outcome measurement is likely to be influenced by lack of

blinding; Blinding of key study participants and personnel attempted, but likely that the blinding could have been broken; Either participants or some key study personnel were not blinded, and the non-blinding of others likely to introduce bias.

* This global rating is challenging. May want to omit and use only the ratings below.

3.a) Were patients blinded?

Definitely yes Probably yes Probably no Definitely no

3.b). Were healthcare providers blinded?

Definitely yes Probably yes Probably no Definitely no

3.c). Were data collectors blinded?

Definitely yes Probably yes Probably no Definitely no

3.d). Were outcome assessors blinded?

Definitely yes Probably yes Probably no Definitely no

3.e). Were data analysts blinded?

Definitely yes Probably yes Probably no Definitely no

1. **Was loss to follow-up (missing outcome data) infrequent?**

Definitely yes Probably yes Probably no Definitely no

(low risk of bias) (high risk of bias)

Examples of low risk of bias: No missing outcome data; Reasons for missing outcome data unlikely to be related to true outcome (for survival data, censoring unlikely to be introducing bias); Missing outcome data balanced in numbers across intervention groups, with similar reasons for missing data across groups; For dichotomous outcome data, the proportion of missing outcomes compared with observed event risk not enough to have a important impact on the intervention effect estimate; For continuous outcome data, plausible effect size (difference in means or standardized difference in means) among missing outcomes not enough to have an important impact on observed effect size; Missing data have been imputed using appropriate methods.

Examples of high risk of bias: Reason for missing outcome data likely to be related to true outcome, with either imbalance in numbers or reasons for missing data across intervention groups; For dichotomous outcome data, the proportion of missing outcomes compared with observed event risk enough to induce important bias in intervention effect estimate; For continuous outcome data, plausible effect size (difference in means or standardized difference in means) among missing outcomes enough to induce clinically relevant bias in observed effect size; ‘As-treated’ analysis done with substantial departure of the intervention received from that assigned at randomization; Potentially inappropriate application of simple imputation.

1. **Are reports of the study free of suggestion of selective outcome reporting?***

Definitely yes Probably yes Probably no Definitely no

(low risk of bias) (high risk of bias)

Examples of low risk of bias: The study protocol is available and all of the study’s pre-specified (primary and secondary) outcomes that are of interest in the review have been reported in the pre-specified way; The study protocol is not available but it is clear that the published reports include all expected outcomes, including those that were pre-specified (convincing text of this nature may be uncommon).

Examples of high risk of bias: Not all of the study’s pre-specified primary outcomes have been reported; One or more primary outcomes is reported using measurements, analysis methods or subsets of the data (e.g. subscales) that were not pre-specified; One or more reported primary outcomes were not pre-specified (unless clear justification for their reporting is provided,

such as an unexpected adverse effect); One or more outcomes of interest in the review are reported incompletely so that they cannot be entered in a meta-analysis; The study report fails to include results for a key outcome that would be expected to have been reported for such a study

* This item sufficiently difficult to judge that may be omitted.

1. **Was the study apparently free of other problems that could put it at a risk of bias?***

Definitely yes Probably yes Probably no Definitely no

(low risk of bias) (high risk of bias)

Examples of low risk of bias: The study appears to be free of other sources of bias.

Examples of high risk of bias: Had a potential source of bias related to the specific study design used; Stopped early due to some data-dependent process (including a formal-stopping rule); Had extreme baseline imbalance; Has been claimed to have been fraudulent; Had some other problem.

* May omit this item.

Table S1. List of excluded articles with reasons

| **Year** | **First Author** | **Journal/Conference** | **Reason for exclusion (References provided is for the most recent publication of the trial)** |
| --- | --- | --- | --- |
| **Bryan Disc** | | | |
| 2008 | Anderson[[36](#_ENREF_36)] | Spine | Patient overlap[[5](#_ENREF_5)] |
| 2006 | Coric[[37](#_ENREF_37)] | J Neurosurg: Spine | Patient overlap[[5](#_ENREF_5)] |
| 2010 | Garrido[[38](#_ENREF_38)] | J Spinal Disord Tech | Patient overlap[[5](#_ENREF_5)] |
| 2005 | Hacker[[39](#_ENREF_39)] | J Neurosurg: Spine | Patient overlap[[5](#_ENREF_5)] |
| 2009 | Heller[[8](#_ENREF_8)] | Spine | Patient overlap[[5](#_ENREF_5)] |
| 2008 | Peng-Fei[[40](#_ENREF_40)] | International Orthopaedics | Non-consecutive patients |
| 2011 | Sasso[[41](#_ENREF_41)] | Spine | Patient overlap[[5](#_ENREF_5)] |
| 2008 | Sasso[[42](#_ENREF_42)] | J Spinal Disord Tech | Patient overlap[[5](#_ENREF_5)] |
| 2007 | Sasso[[43](#_ENREF_43)] | J Spinal Disord Tech | Patient overlap[[5](#_ENREF_5)] |
| 2007 | Sasso[[44](#_ENREF_44)] | Spine | Patient overlap[[5](#_ENREF_5)] |
| 2009 | Xu[[45](#_ENREF_45)] | Spine | Non-consecutive patients. NDI and SF-36 collected but not reported. Authors were contacted but did not reply. |
| **Kineflex-C** | | | |
| 2008 | Guyer[[46](#_ENREF_46)] | Spineweek 2008 | Patient overlap[[6](#_ENREF_6)] |
| **Mobi-C** | | | |
| 2011 | Davis[[47](#_ENREF_47)] | NASS 2011 conference | No single level arthroplasty |
| 2011 | Hisey[[48](#_ENREF_48)] | Eurospine 2011 conference | Could not contact authors. Only abstract available. |
| 2011 | Nunley[[49](#_ENREF_49)] | NASS 2011 conference | Contacted author but no reply received. Only abstract available. |
| **PCM** | | | |
| 2011 | Park[[50](#_ENREF_50)] | Spine | No patient important outcomes reported. |
| 2011 | Howell[[51](#_ENREF_51)] | NASS 2011 conference | Patient overlap[[21](#_ENREF_21)] |
| **Prestige** | | | |
| 2007 | Mummaneni[[9](#_ENREF_9)] | J Neurosurg: Spine | Patient overlap[[23](#_ENREF_23)] |
| 2004 | Porchet[[52](#_ENREF_52)] | Neurosurg Focus | Patient overlap[[23](#_ENREF_23)] |
| 2008 | Riina[[53](#_ENREF_53)] | Am J Orthop | Patient overlap[[23](#_ENREF_23)] |
| **Pro-Disc** | | | |
| 2009 | Anakwenze[[54](#_ENREF_54)] | Spine | Patient overlap[[25](#_ENREF_25)] |
| 2011 | Auerbach[[55](#_ENREF_55)] | Spine | Patient overlap[[25](#_ENREF_25)] |
| 2009 | Murrey[[7](#_ENREF_7)] | The Spine Journal | Patient overlap[[25](#_ENREF_25)] |
| 2007 | Nabhan[[56](#_ENREF_56)] | Eur Spine J | Patient overlap[[57](#_ENREF_57)] |
| 2007 | Nabhan[[58](#_ENREF_58)] | Spine | Patient overlap[[57](#_ENREF_57)] |
| **SECURE-C** | | | |
| 2010 | Marzluff[[59](#_ENREF_59)] | NASS 2010 conference | Could not contact authors. Only abstract available. |
| **Bryan, Kineflex-C and Discover** | | | |
| 2010 | Coric[[60](#_ENREF_60)] | J Neurosurg: Spine | Patient overlap[[7-9](#_ENREF_7)] |
| **Kineflex-C, Mobi-C and Advent Cervical Disc** | | | |
| 2010 | Jawahar[[61](#_ENREF_61)] | The Spine Journal | Patient overlap[[37](#_ENREF_37),[49](#_ENREF_49)] |
| **Multiple prosthesis (undetermined)** | | | |
| 2011 | Utter[[62](#_ENREF_62)] | EANS 2011 conference | Review study |
| **Undetermined** | | | |
| 2008 | Abitbol[[63](#_ENREF_63)] | Spineweek 2008 | Could not contact authors. Only abstract available. |
| 2008 | Fischgrund | Spineweek 2008 | Could not contact authors. Only abstract available. |

Table S2. Risk of bias for the 9 included studies using a modified approach to the Cochrane risk of bias tool

|  | **Risk of bias** | | | | | | | **Trial characteristics** | | |
| --- | --- | --- | --- | --- | --- | --- | --- | --- | --- | --- |
| **Source** | **Random sequence generation** | **Allocation concealment** | **Blinding of participants and personnel** | **Blinding of outcome assessors** | **Infrequent loss to follow-up** | **Free of selective outcome reporting** | **Free of other bias** | **Statistical analysis (Per protocol, intention to treat, etc.)** | **How is loss to follow-up handled?** | **Affiliated with industry** |
| Burkus et al. 2010[[23](#_ENREF_23)] | Probably yes | Probably no | Definitely no | Definitely no | Probably yes | Probably yes | Probably no | Not mentioned | LOCF | Yes |
| Coric et al. 2011[[6](#_ENREF_6)] | Probably yes | Probably no | Probably no | Probably no | Definitely yes | Definitely yes | Probably no | Not mentioned | Not mentioned | Yes |
| Delamarter et a. 2010[[25](#_ENREF_25)] | Probably yes | Definitely yes | Definitely yes | Definitely yes | Probably yes | Definitely yes | Probably no | Per-protocol | Not mentioned | N/A |
| McAfee et al. 2011* | Probably yes | Probably no | Probably no | Probably no | Probably yes | Probably no | Probably no | Not mentioned | Not mentioned | N/A |
| McAfee et al. 2010[[20](#_ENREF_20)] | Probably yes | Probably no | Probably no | Probably no | Probably yes | Definitely no | Probably no | Not mentioned | Not mentioned | N/A |
| Nabhan et al. 2007[[24](#_ENREF_24)] | Definitely yes | Definitely yes | Probably no | Probably no | Definitely yes | Probably no | Definitely yes | Not mentioned | Ignored | N/A |
| Nabhan et. al 2011[[26](#_ENREF_26)] | Definitely yes | Definitely yes | Probably no | Probably no | Definitely yes | Definitely no | Definitely yes | Not mentioned | Not mentioned | N/A |
| Sasso et. al 2011[[5](#_ENREF_5)] | Probably yes | Definitely yes | Definitely no | Definitely no | Probably yes | Definitely no | Probably no | Per-protocol | LOCF | Yes |
| Wang et. al 2008[[22](#_ENREF_22)] | Probably no | Probably no | Probably no | Probably yes | Definitely no | Probably yes | Probably no | Not mentioned | Ignored | N/A |

Figure S3. Cochrane risk of bias by trial. Green indicates low risk of bias, red indicates high risk of bias.

**
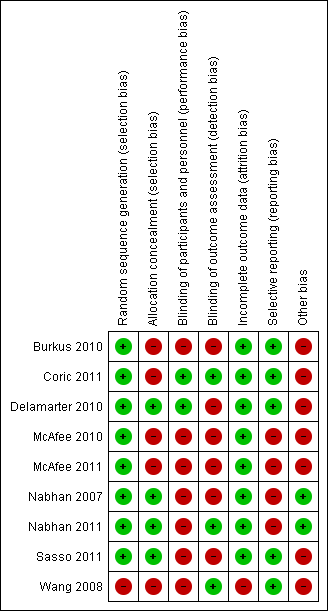
**
